# Training programmes to improve evidence uptake and utilisation by physiotherapists: a systematic scoping review

**DOI:** 10.1186/s12909-018-1121-6

**Published:** 2018-01-15

**Authors:** Jessica Stander, Karen Grimmer, Yolandi Brink

**Affiliations:** 1 Division of Physiotherapy, Faculty of Medicine and Health Sciences, Stellenbosch University, Francie van Zijl Drive, Tygerberg, Cape Town, 7505 South Africa; 2 International Centre for Allied Health Evidence (iCAHE), City East Campus, P4-18 North Terrace, University of South Australia, Adelaide, 5000 Australia

**Keywords:** Clinical practice guidelines, Evidence-based practice, Knowledge translation, Physiotherapy

## Abstract

**Background:**

Research training programmes are a knowledge translation (KT) intervention which aim to improve research evidence uptake by clinicians. Whilst KT training programmes have been reported to significantly improve evidence uptake by physiotherapists, it is unclear which aspects of training optimally assist KT into physiotherapy practice. The purpose of the review was to establish the body of evidence regarding KT training programmes to improve physiotherapists’ use of evidence-based practice (EBP) and clinical practice guidelines (CPG).

**Methods:**

A systematic scoping review was undertaken in line with the adapted Arksey and O’Malley framework. Nine electronic databases (CINAHL, BIOMED CENTRAL, Cochrane, Web of Science, PROQUEST, PUBMED, OTseeker, Scopus, ERIC) were searched. Targeted keywords identified primary research articles of any hierarchy, that described the nature and impact of KT training programmes for physiotherapists. Where systematic reviews were identified, the component primary studies were considered individually for relevance. Critical appraisal was not undertaken due to the nature of a scoping review, and data was reported descriptively.

**Results:**

Ten systematic reviews were identified (yielding four relevant primary studies). Five additional primary studies were identified (two randomised controlled trials, two non-randomised controlled trials and one pre-post study) which were not included in the original systematic reviews. This provided nine eligible primary research studies for review. The KT strategies were all multi-faceted. Interactive sessions, didactic sessions, printed material and discussion and feedback were consistently associated with effective outcomes. When KT strategies addressed local barriers to EBP utilisation, there were better success rates for EBP and CPG uptake, irrespective of the outcome measures used. There were no consistent ways of measuring outcome.

**Conclusion:**

Multi-faceted KT strategies designed to address local barriers to knowledge translation were most effective in improving EBP/ CPG uptake among physiotherapists.

**Electronic supplementary material:**

The online version of this article (doi: 10.1186/s12909-018-1121-6) contains supplementary material, which is available to authorized users.

## Background

Evidence-based practice (EBP) occurs when there is integration between clinicians’ clinical decision-making ability and skills, the best available research evidence, and patients’ choices and beliefs. There is increasing recognition of the need to align EBP with local contexts [1, 2]. Knowledge translation (KT) activities are designed to facilitate clinician awareness research findings into the hands of end-users, therefore KT underpins uptake of EBP. KT is defined as the evaluation, synthesis and application of local and global research evidence into formats acceptable by patients, practitioners and other stakeholders, to inform best management decisions for patients [3–5].

KT intervention studies for physiotherapists were first reported in 1999 [6]. They focussed on passive approaches such as clinical practice guideline (CPG) handouts (in hard copy format or electronically via email) [6]. Over the last 17 years, KT strategies have become increasingly multi-faceted and methodologically robust and have been underpinned by increasingly sophisticated theoretical frameworks [3]. KT intervention strategies have been shown to be more effective when local barriers and facilitators to evidence implementation are identified and clearly outlined [3, 7]. Research suggests that practice-based knowledge and research-based knowledge should be integrated to ensure the most effective and ethical assessment, and management of patients [8, 9].

The literature indicates that physiotherapists generally have positive attitudes towards implementing evidence into practice [10, 11]. However, recent systematic reviews have identified consistent barriers to implementation of best evidence-based care, including lack of time; lack of resources; lack of support by employers; lack of skills and understanding of EBP theory and application; lack of interest, and a perception of poor generalizability of research evidence to local contexts [11]. Similar barriers to EBP use have been reported for physiotherapists across developing and developed countries [12, 13].

### Putting CPGs into practice

Clinical practice guidelines (CPGs) were described by Treweek et al (2013) as ‘*a convenient way of packaging evidence and presenting recommendations to healthcare decision makers*‘ (p.2) [14]. There is an increasing number of readily-available, high-quality CPGs for clinicians, managers, policy makers and patients. These can assist in shared decision-making between healthcare professionals and patients [15]. It is believed that adherence to CPGs improves quality of care, health and service outcomes, and improve cost-benefits [16, 17]. CPGs may also have a role in decreasing the use of low value healthcare options [16–18]. CPG use by physiotherapists is in its early stages globally, and physiotherapists may not necessarily adopt CPG recommendations if they are unaware of the processes by which the recommendations were derived, and/ or if recommendations are contrary to local beliefs, practices and resources [19]. EBP training is a foundational skill for CPG development and/ or uptake [20]. If it is assumed that using CPGs in clinical physiotherapy practice will improve health, cost and service outcomes, then improving CPG uptake by physiotherapists will require effective tailored KT approaches, which address local barriers to CPG implementation [21, 22].

### Behaviour change and learning theories

A well-designed KT conceptual or theoretical framework can help guide the researcher in the design and implementation of KT strategies and ultimately to evaluate its effectiveness [23, 24]. Many behaviour change theories have been proposed, many with overlapping elements [23]. There is no standard approach. However, a commonly used theory is Grol’s 5-step Implementation of Change model [23, 25]. This has comparable domains to the Knowledge-to-Action and Promoting Action on Research Implementation in Health Services (PARiHS) frameworks [23, 25]. Developed from a comprehensive overview of behaviour change theories, Grol’s Implementation of Change model is purported to assist in identifying the domains that must be addressed during a KT intervention, using a structured, sequential manner to improve its chances of success [26]. An essential element in these behaviour change approaches is the importance of considering local contexts, and barriers for uptake of evidence.

There has also been a body of work investigating how adult healthcare professionals, including allied healthcare professionals, learn best [27, 28]. Allied health is an umbrella term, which encompasses different disciplines (including physiotherapists) with different tasks, training, competencies and learning styles [27]. There is no single learning theory that is applicable to all adults, in this case, clinicians [29]. Understanding adult learning theories can help educators use the most appropriate learning theories to meet the learning styles of the class. Considering KT training programmes, well-thought-out adult learning theories relevant to specific adult learners’ needs, and contextualised to local barriers to uptake, could underpin the development of effective multi-faceted KT interventions [30].

### Outcomes of KT interventions

Evaluation of attitudes and behaviours towards EBP has not been as well researched as evaluation of knowledge and skills regarding the use of EBP [25]. There is a recognised gap between what healthcare professionals know, and what they actually do [22, 24, 25]. Scurlock-Evans et al. (2014) emphasised the need for further research to better understand why this gap occurs [10]. These authors also suggest the development of sensitive measures supporting the investigation of differences between physiotherapists’ attitudes, behaviours, knowledge and skills regarding EBP implementation in their daily practice behaviours [10].

This systematic scoping review was undertaken as a preliminary step in developing a tailored KT training programme for physiotherapists working in primary care in South Africa. There is no history in South Africa of educating physiotherapists about CPG use. South Africa is a land of great contrast, with a population of approximately 56 million people. Physiotherapists are few and far between in rural and remote communities, a scarcity that is particularly relevant when compared to local population needs [31, 32]. CPGs are needed to optimise care delivery, efficiency and effectiveness and to significantly impact on the burden of disease and disability [31, 32].

The purpose of this review was to establish the body of evidence regarding KT training programmes to improve physiotherapists’ use of EBP and CPGs.

## Methods

### Strategies

The overall purpose of the review was addressed by:

1. Identifying which underlying theories and models of behaviour change were reported as being relevant to the development of the included KT training programmes
2. Identifying and describing the intervention elements in the training programmes;
3. Describing the outcome measures reported in the training programmes; and
4. Mapping the elements of the training programmes against evidence of their effectiveness.

### Design

A systematic scoping review was conducted. A scoping review is a systematic search, literature evaluation, and qualitative and quantitative descriptive synthesis of current research evidence for a broad topic [33]. Scoping reviews generally aim to identify the volume, type and focus of current research, and gaps in current evidence [33]. It also aims to identify the types of study designs applied to answer specific types of research questions [33].

### Quality framework and reporting standard

An adapted Arksey and O’Malley scoping review framework was followed [34, 35]. This provided a structured review process to ensure that complexities of the current body of evidence were explored, and gaps in the body of literature were identified [34]. The adapted framework included the following steps: 1) formulating the research questions; 2) identifying relevant studies; 3) study selection; 4) charting the data; and 5) collating, summarising and reporting the results [34, 35].

### Inclusion criteria

Only papers published in English and available in full-text format were included. Any prospective comparative study was eligible for inclusion if it reported within-group and/ or between group comparisons. Thus, included designs could be pre-post (where subjects acted as their own controls); quasi-controlled studies (historical controls or time-series studies, including cohort studies); controlled clinical trials or randomised controlled trials.

### Exclusion criteria

Studies were excluded if they were only available in abstract format or formed part of published conference proceedings. Studies were excluded if they did not report on the elements of the training programme, and/or did not specifically report on physiotherapists’ knowledge, skills, attitudes and/ or behaviour outcomes. Retrospective studies were excluded, as were prospective studies that did not report on within- or between-group change.

### Search framework

A PICO or PIO framework was proposed, to account for the comparative studies that had, or did not have, a comparison group.

P = Physiotherapists (or allied health information from which physiotherapy data could be extracted).

I = Any training programme which aimed to improve physiotherapists’ knowledge, skills, attitudes and/ or behaviour regarding EBP or CPGs. The interventions were described by their intent, relevant to EBP or CPGs. This broad inclusion criteria reflected the possible timeframes over which the training programmes were presented, and the outcomes may be measured.

C = Any comparator, or none.

O = Any outcome measure that reported directly on the physiotherapists’ knowledge, skills, attitudes and/ or behaviour as a result of the KT interventions. We were mindful that not all of these outcomes are viably measured immediately after training. For instance, knowledge and skills can appropriately be measured over a short timeframe immediately after the training intervention, and realistically could be measured again at a later stage to investigate maintenance of knowledge and skills. However, attitudes and behaviour change may take longer to occur, and thus may be most appropriately measured at some period after the training programme has been completed.

### Search terms

The search terms were broad and included: (“clinical practice guidelines” OR “guidelines”) AND (physiotherapist OR physiotherapy OR physical therapy OR physical therapist) AND (“strategies” OR “interventions”) AND “effect”. The search was conducted using Medical Subject Headings (MeSH) where applicable to ensure all possibly relevant articles would be obtained. The MESH terms included “allied health occupations” and “physical therapists”. Table 1 outlines an example of the search strategy used.

**Table 1:** Search strategy example

	Search strings
#1	(“clinical practice guidelines” OR “guidelines”) AND (“strategies” OR “interventions”) AND “effect” AND “Allied Health Occupations”[Mesh] Filters: English
#2	(“physical therapists”[MeSH Terms] OR “physical therapists”[All Fields] OR “physiotherapists”[All Fields]) AND (“clinical practice guidelines” OR “guidelines”) AND (“strategies” OR “interventions”) AND “effect” Filters: English

### Data bases searched

Nine electronic databases (CINAHL, BIOMED CENTRAL, Cochrane, PEDro, PROQUEST, PUBMED, OTseeker, Scopus, ERIC) were searched from inception to June 2017.

### Hierarchy of evidence

This was determined according to the relevant National Health and Medical Research Council (NHMRC) hierarchy of intervention evidence [36].

### Quality appraisal

No quality appraisal of the included studies was undertaken, as is usual in a scoping review [33]. The hierarchy of evidence provided a framework by which potential bias could be assessed.

### Classification

The interventions used in the studies were classified using the Effective Practice and Organisation of Care (EPOC) taxonomy of implementation strategies as professional interventions [37]. The EPOC taxonomy was also used as a form of checklist by which the studies were assessed for component implementation strategies [37]. Knowledge translation elements were extracted from, and defined according to, the EPOC taxonomy, or referred to in the manner of the individual studies [37].

### Analysis

Data was extracted regarding study characteristics (country of origin, year of publication, number of subjects, study design and length of time over which outcomes were measured). A second descriptive table reported on the elements of each training programme and the outcome measures used. Common elements of training programmes and common outcome measures were identified. Descriptive tables were constructed to provide information from the papers that reported significant changes in outcome measures and where possible, these were linked to elements of the training programmes.

### Decision making

One reviewer (JS) and a health sciences librarian searched the electronic databases. All three authors (JS, YB, KG) independently applied all inclusion and exclusion criteria. JS identified potentially eligible articles by screening all titles, reading the abstracts and determining initial eligibility. All authors read the full text article of potentially eligible studies and determined final study inclusion. Any concerns on article inclusion were discussed among the reviewers a final decision [35].

## Results

### Eligible papers

**Fig. 1:**
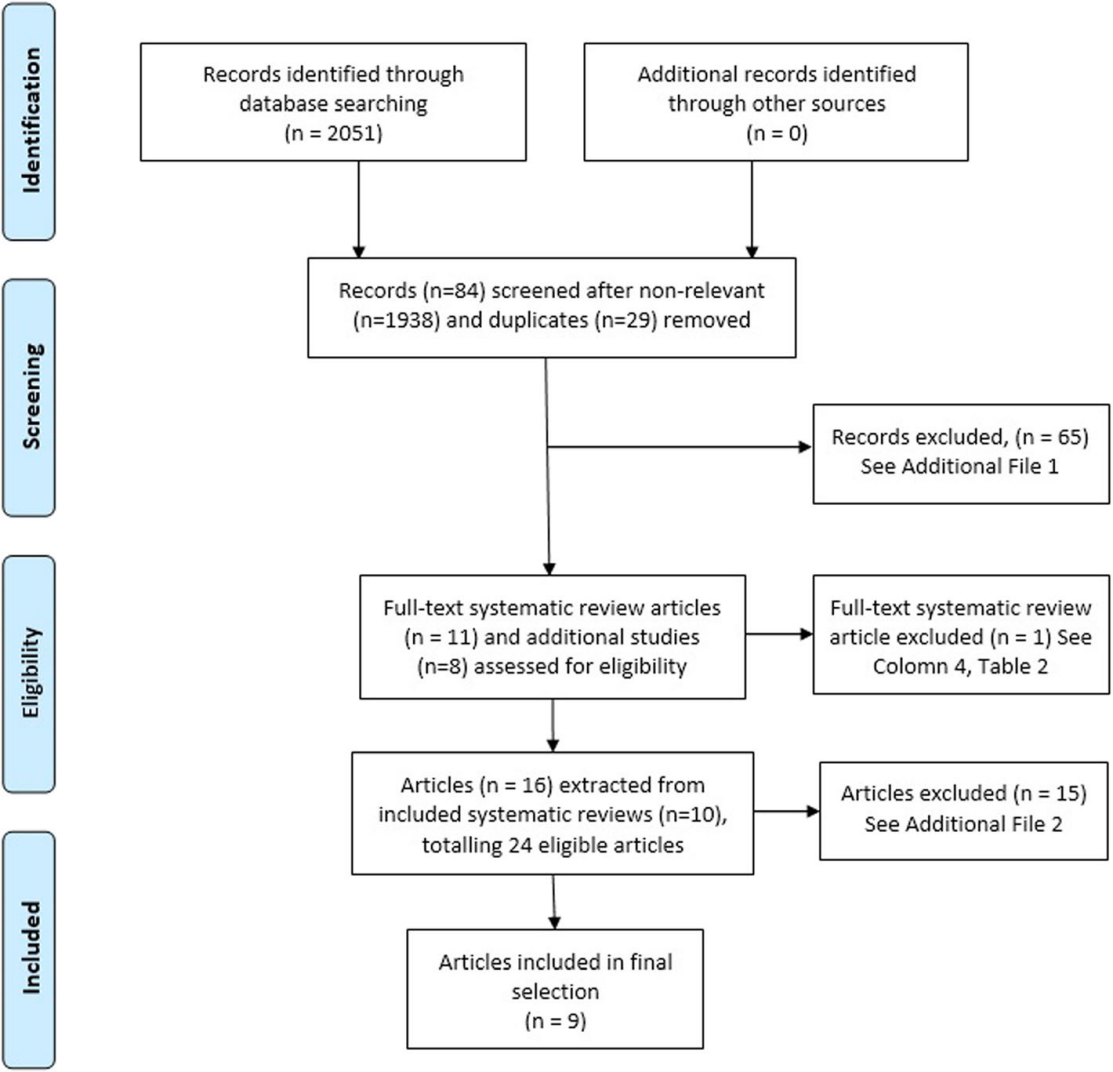
PRISMA flow diagram

After full text screening, eleven systematic reviews (SRs), and eight primary studies not reported in the SRs were identified as being potentially relevant. One systematic review [22] was subsequently excluded due to not including any studies specifically about physiotherapists. Figure 1 reports on the PRISMA flow diagram of article selection and inclusion [38].

Table 2 outlines the process of inclusion and exclusion of the systematic reviews. Information on excluded articles is provided in supplementary material (Additional files 1 and 2).

**Table 2:** Inclusion and exclusion of systematic reviews reviewed by full text

	Baker et al. 2015	Hecht et al. 2016	Johnson & May 2015	Jones et al. 2015	Menon et al. 2009	Prior et al. 2008	Scott et al. 2012	Scurlock-Evans et al. 2014	Van der Wees et al. 2008	Hakkennes & Dodd 2008	Dizon et al. 2012
Elements/ strategies of PT training programmes for EBP utilisation?	✓	✓	✗	✓	✓	✗	✓	✓	✗	✗	✓
Elements/ strategies of PT training programmes for CPG utilisation?	✗	✗	✗	✗	✗	✓	✗	✗	✓	✓	✗
Only reporting on PT?	✗	✗	✗	✗	✗	✗	✗	✓	✓	✗	✗
Effectiveness of these training programmes?	✓	✓	✗	✓	✓	✓	✓	✓	✓	✓	✓
Outcome measures reported on for training programmes?	✓	✓	✗	✓	✓	✓	✓	✗	✓	✓	✓
Included in scoping review?	✓	✓	✗	✓	✓	✓	✓	✓	✓	✓	✓

Key: *PT* Physiotherapy, *EBP* evidence-based practice, *CPGs* clinical practice guidelines

### Subsequent study identification

Not all the studies within the SRs studied KT activities specifically for physiotherapists. Thus, the component studies which reported on physiotherapists were extracted for the scoping review data set. From the ten identified systematic reviews [10, 39–47], four primary component studies were identified as relevant to the review. Combined with the additional five primary articles identified in the search, this totalled nine relevant primary research articles for the review. All nine articles provided relevant information on KT strategies for targeted training in EBP and CPGs for physiotherapists. Table 3 maps the number of times each article was reported on in the different systematic reviews. Table 3 also highlights that five of the included articles were not reported on in any of the systematic reviews that were found. Only studies reporting on training in the principles of EBP and CPG use was included in this review.

**Table 3:** Mapping of final included articles to systematic reviews

SR / Article [Author (year), country]	Baker et al. 2015	Hecht et al. 2016	Jones et al. 2015	Menon et al. 2009	Prior et al. 2008	Scott et al. 2012	Scurlock-Evans et al. 2014	Van der Wees et al. 2008	Hakkennes & Dodd 2008	Dizon et al. 2012	Total times reported /10
Bekkering et al (2005), the Netherlands [51]			✓	✓		✓		✓	✓		5
Dizon et al (2014), the Phillipines [48]	✓	✓									2
Rebbeck et al (2006), Australia [49]			✓	✓		✓		✓	✓		5
Stevenson et al (2004), United Kingdom [50]		✓		✓		✓		✓		✓	5
Maas et al (2015), the Netherlands [52]											0
Van Dulmen et al (2014), the Netherlands [53]											0
Bernhardsson et al (2014) Sweden [54]											0
Olsen et al (2015), Norway [55]											0
Tilson et al (2014), USA [56]											0
Total reported physiotherapy articles per systematic review	1	2	2	3	0	3	0	3	2	1	

### Hierarchy of evidence and sample description

Table 4 outlines the study design of each included study. Six studies were level II (randomised controlled trials (RCTs)) [48–53] and three studies were level III-2 (non-randomised controlled trials and pre-post studies) [54–56]. The RCTs and non-RCTs comprised 463 and 306 total participants respectively. The mixed methods pre-post study comprised of 18 participants.

**Table 4:** Sample description

Author	Study design	Sample size
Bekkering et al (2005), the Netherlands [51]	Cluster RCT	113
Dizon et al (2014), the Phillipines [48]	RCT	54
Rebbeck et al (2006), Australia [49]	RCT	27
Stevenson et al (2004), United Kingdom [50]	RCT	30
Maas et al (2015), the Netherlands [52]	Cluster RCT	149
Van Dulmen et al (2014), the Netherlands [53]	Cluster RCT	90
Bernhardsson et al (2014) Sweden [54]	Non-RCT	277
Olsen et al (2015), Norway [55]	Non-RCT	29
Tilson et al (2014), USA [56]	Mixed methods Before-after study	18

Key: *RCT* Randomised controlled trial

### Study focus

Table 5 outlines the study focus. Five studies were focussed on CPGs [49, 51–54] and the other four studies were focussed on EBP [48, 50, 56]. Whilst similar data was extracted from all studies, the different foci (i.e. CPGs versus EBP) were addressed by reporting the data separately.

**Table 5:** Mapping of Interventions

		EPOC			Knowledge Translation Elements									Local barriers/ contexts addressed Yes / No
Author	Focus	PE Materials	PE Meetings	PE Outreach	Didactic sessions	Interactive sessions	Printed Material	Discussion & Feedback	Reminders	Role-play	Online Support	Opinion Leaders	Peer Assessment	
KT strategy used		P	A	A	P	A	P	A	A	A	A	A	A	
Bekkering et al (2005), the Netherlands [51]	CPG	✓	✓		✓	✓	✓	✓	✓	✓				Yes
Dizon et al (2014), the Phillipines [48]	EBP	✓	✓	✓	✓	✓	✓	✓	✓		✓			Yes
Rebbeck et al (2006), Australia [49]	CPG	✓	✓	✓	✓	✓	✓		✓			✓		Yes
Stevenson et al (2004), United Kingdom [50]	EBP	✓	✓		✓	✓		✓				✓		Yes
Maas et al (2015), the Netherlands [52]	CPG	✓	✓			✓	✓	✓		✓			✓	Yes
Van Dulmen et al (2014), the Netherlands [53]	CPG	✓	✓			✓	✓	✓		✓			✓	Yes
Bernhardsson et al (2014) Sweden [54]	CPG	✓	✓	✓	✓	✓	✓		✓		✓			Yes
Olsen et al (2015), Norway [55]	EBP	✓	✓		✓	✓		✓	✓					No
Tilson et al (2014), USA [56]	EBP	✓	✓		✓	✓	✓	✓			✓			Yes
Total:		9	9	3	7	9	7	7	5	3	3	2	2	

Key: *EPOC* EPOC classification, *PE* Professional Education, *P* passive strategy, *A* active strategy, *CPG* Clinical practice guideline, *EBP* evidence-based practice

### Classifications of components of training programmes

Nine elements of KT interventions were identified from the included studies. All studies utilised multi-faceted KT interventions, incorporating both passive and active strategies in delivering the programmes [37]. Table 5 also outlines the intervention elements. All studies used interactive sessions. Didactic sessions, printed materials and discussion and feedback were used in seven out of the nine studies [48–51, 54, 56]. Reminders were used in five studies [48, 49, 51, 54, 55]. Role-play was used in three studies [51–53], as was online support [48, 54, 56]. Opinion leaders were used in two studies [49, 50], as was peer assessment [52, 53].

### Inclusion of local barriers and contexts

This review found evidence of how local barriers and contexts were addressed in the interventions (Table 5). Only Olsen et al [55] failed to report that they based their programme on local barriers to change and reported that barriers to EBP use may be the reason why change was not sustained. Four studies [51, 53, 54, 56] identified local barriers to EBP or CPG use as part of designing their training programme and this may have assisted them to contextualise the programme for their chosen physiotherapy community.

### Underlying theories/ models of behaviour change and description of interventions

Table 6 reports on the different underlying theories/ models of behaviour change. Grol’s Implementation of Change model [23, 25] was used in two studies [51, 54], albeit using different references to this work. Tilson et al [56] utilised Graham’s knowledge-to-action and PARiHS frameworks [23]. Bandura’s social cognitive theory and the adult learning theory was also used by Tilson et al [56], and by Dizon et al [48]. Four studies did not report any underlying theory or model for their intervention [49, 50, 53, 55]. Table 6 also summarises the duration and types of interventions used by each study.

**Table 6:** Description of intervention

Author	Underlying theories	Duration of intervention
Bekkering et al (2005), the Netherlands [51]	IoC model	2x 2.5-h sessions
Dizon et al (2014), the Phillipines [48]	ALT, educational strategies	1x one-day session, continues support 3-months post-intervention
Rebbeck et al (2006), Australia [49]	None reported	1x one-day session, educational outreach (2-h) 6-months post-intervention
Stevenson et al (2004), United Kingdom [50]	None reported	1x 5-h session
Maas et al (2015), the Netherlands [52]	SCT, Social constructivist, stages of change & SDL theory	4x 3-h sessions
Van Dulmen et al (2014), the Netherlands [53]	None reported	4x2-hour sessions over 6-months
Bernhardsson et al (2014) Sweden [54]	IoC model	1x 3-h session
Olsen et al (2015), Norway [55]	None reported	4x half-day sessions
Tilson et al (2014), USA [56]	SCT, ALT, PARiHS, KtA framework	1x 2-day session, with continued small group work over 6-months

Key: *RCT* Randomised controlled trial, *IoC* Implementation of Change, *ALT* Adult learning theory, *SCT* Social cognitive theory, *SDL* Self-directed learning, *PARiHS* Promoting Action on Research Implementation in Health Services, *KtA* Knowledge-to-action

### Outcomes and evidence for effectiveness

The reported outcomes are summarised in Table 7. Outcome measures included adherence/ behaviour, knowledge, skills, attitudes and beliefs, awareness, attainment of goals and reflective practice. The most commonly reported outcome was adherence/ behaviour in all but two studies [48, 49, 51–54, 56]. Adherence to guidelines was referred to as “guideline-consistent practice” by two studies [49, 53]. Knowledge was the next most commonly reported measure in six studies [48, 49, 53–56]. Attitudes and beliefs were measured in five studies [48, 50, 54–56], and skills were measured in four studies [48, 54–56]. Awareness was measured in two studies, but was described differently by each: awareness of performance [52] versus awareness of CPGs [54]. Attainment of goals and reflective practice were only measured in one study [52]. Short term refers to measurement intervals of less than three months, while long term refers to intervals of equal or more than three months.

**Table 7:** Mapping of Outcomes

Author	Focus	Adherence / Behaviour	Knowledge	Skills	Attitudes & Beliefs	Awareness	Attainment of Goals	Reflective Practice	Measurement Interval
Bekkering et al (2005), the Netherlands [51]	CPG	↑							Baseline, 1 month
Dizon et al (2014), the Phillipines [48]	EBP	↑ (LT)	↑ (ST, LT)	↑ (ST, LT)	↑ (ST, LT)				Baseline, 3 months
Rebbeck et al (2006), Australia [49]	CPG	↑ (ST, LT)	↑ (ST, LT)						Baseline, 12 months
Stevenson et al (2004), United Kingdom [50]	EBP	−			↑ (ST, LT)				Baseline, 6 months
Maas et al (2015), the Netherlands [52]	CPG	↑ (LT)				↑ (LT)*	↑ (LT)	-	Baseline, 6 months
Van Dulmen et al (2014), the Netherlands [53]	CPG	↑	↑						Baseline, 6 months
Bernhardsson et al (2014) Sweden [54]	CPG	↑	↑	↑	-	↑^			Baseline, 6 months
Olsen et al (2015), Norway [55]	EBP	-	↑	↑	↑				Baseline, 6 months
Tilson et al (2014), USA [56]	EBP	↑ (LT)	-	-	↑				Baseline, 6 months
Total studies:		7/9	5/6	3/4	4/5	2/2	1/1	0/1	

Key: ↑ statistically significant improvement; − = non-significant change; □ = not reported; * = performance; ^ = clinical practice guidelines

*ST* short term, *LT* long term

Table 7 also reports change in the study outcomes as a result of interventions. Seven studies [48, 49, 51–54, 56] reported improvement and two studies [51, 55] reported no change in adherence/ behaviour. Five studies [49, 50, 53–55] reported improvement and one study [56] reported no change in knowledge. Three studies each reported improvement in skills [49, 54, 55] and attitudes and behaviours [49, 51, 55, 56]. One study each reported no change in skills [56] or attitudes and behaviours [54] Only one study each assessed for and found an improvement in awareness of performance [52] and awareness of CPGs [54]. Attainment of goals were found to improve in the one study [52] that evaluated it. Reflective practice was found to stay unchanged in the one study [52] that evaluated it.

## Discussion

This review is the first that we are aware of, that dissects the elements of KT training programmes provided for EBP and/ or CPGs to physiotherapists, and to relate these to outcome measures, local context issues / barriers, and favourable change in outcomes. The predominant study design used was RCTs. However, taking into consideration that the participants’ view may affect the design, execution and ultimate effect of the training, a mixed methods design may potentially benefit a study. The review found that multi-faceted strategies were most commonly associated with significant changes in learning outcomes, with adherence/ behaviour change to CPGs being the most commonly reported outcome. The review also identified that there was no one consistent way of educating PTs, or in measuring the effectiveness of KT for either EBP, or CPG, outcomes. Identifying local barriers to EBP or CPG use may assist in contextualising the training programme for the chosen physiotherapy group.

### Underlying theories/ models of behaviour change

The KT training programmes were underpinned by behaviour change models and learning theories, and it was not clear which theory underpinned the programme that would most likely lead to effective training. However, the training programmes not based on a theory or a learning style model appeared, to be less effective than those that were, and this is in line with other literature [3, 24]. This may be due to the fact that a programme, without an underlying theoretical framework, might be of lesser quality and therefore less effective. It seems that further research is required into how specific elements of behaviour change and adult learning that relate to physiotherapists and their practice can be built into KT training programmes [24]. Just as the learning style theorists indicate that there is no ‘one size fits all’ for medical education, this is equally applicable to allied health. Physiotherapists for instance, are known to learn differently to other allied health professionals [28, 57–59]. Thus, it is important for educators to understand not only the most relevant behaviour change theory(ies) for different allied health disciplines they teach, but to provide training in the most appropriate manner for their audience and the local contexts/ barriers to uptake of EBP [27, 28].

### Elements of training programmes

There was no consistency in elements of training programmes, however, multi-faceted programmes which included at least five different elements appeared to be more effective in producing significant learning outcomes than programmes with fewer elements. This again is consistent with the literature [3, 41]. Our review highlighted the importance of presenting information in different ways, which potentially maps to recipients’ varied ways of learning, and the contexts within which learning occurs [3, 29]. In particular, local barriers and contexts can be addressed by interactive sessions, and discussion and feedback aspects within the KT training programmes. This means that participants can actively identify and consider the issues that may constrain their uptake of evidence into practice, whilst they are in the training programme. Sharing this information with others during training, and having educator-facilitated discussions, could assist in identifying solutions to local context issues, rather than this being an isolating exercise for students once they return to their local practice settings.

Whilst seven studies in our review applied the elements of discussion and feedback in their training programmes, all but two applied these elements within the structured training time. The remaining two studies used a longer-term application of ‘training’ in terms of completion of an activity diary [48, 51]. This could act as a reminder of changed practice, provided a form of feedback for the therapists themselves, their colleagues, and between therapists and educators, as well as assisting trainees to reflect actively on the context and local barriers to implementing EBP. Whereas, diagnostic- and treatment-focussed role-play sessions based on CPG recommendations can potentially facilitate the implementation of CPG into daily practice [51–53].

As part of a multi-faceted KT intervention, reminders (in the form of patient information brochures, information cards for use during episodes of care or emails) may assist in encouraging physiotherapists to utilise EBP and specifically CPGs on a more continuous basis [48, 49, 51, 54, 55]. If required, support via telephone or online regarding the CPGs or EBP utilisation, may also enhance CPG utilisation [48, 54, 56]. These strategies also offer ways in which KT strategies might address local barriers to EBP and CPG uptake.

### Outcomes

It was surprising that skills and knowledge were not the most commonly reported outcomes in the included papers. The authors had assumed that skills and knowledge may be of importance because they have been integral outcomes in KT training programmes since inception [7]. Not only did this scoping review find that all nine included studies assessed adherence/ behaviour change (which requires time elapsed to demonstrate it), but it also found that seven of these studies demonstrated a significant increase in adherence/ behaviour change as a result of the intervention. This finding was encouraging because behaviour change and compliance with EBP principles and CPG recommendations are critical success factors for longer term sustainable outcomes from training [21]. Of note however, was that only two of the included studies actively continued the training intervention after cessation of the formal training programme (both using an activity diary) [48, 51]. It was not possible to tell from this review whether the activity diary used, impacted on behaviours more than simply the time elapsed since training, as in the other seven studies. If this finding is indeed to be believed, it suggests that significant improvements in behaviours and compliance could be anticipated at least three months post training. In our circumstances, this would be conditional upon applying a multi-faceted training programme contextualised to South African physiotherapists in primary care, with, or without, the use of an activity diary.

### Elements of training associated with outcomes

It appears to be a relationship between favourable outcomes for physiotherapy adherence/ behaviour (s) and at least five elements of active programme delivery (interactive sessions, printed material, didactic sessions, discussion and feedback). On this basis, we propose that active KT approaches are more effective than passive KT approaches, such as educational meetings and materials. This may particularly be found when active strategies have multiple components, and when clinicians are able to contribute to the process [7, 41–43] (as in discussion and feedback, or interactive sessions). Passive, single strategy studies reported non-significant outcomes, with dissemination of CPGs through leaflets being least effective [3, 39, 41]. However, CPG dissemination embedded as part of a multi-faceted approach, may increase in its effectiveness [22]. Educational meetings and outreach strategies, reminders and audit and feedback offer better end-results than persuasive processes, such as opinion leaders and consensus processes. If the former is used in combination with each other, it can lead to positive behaviour change [3, 22]. The use of opinion leaders and consensus has the advantage of directly addressing local barriers and contexts. If contextualised barriers to EBP uptake and practice change are identified beforehand, and strategies formed to address, there appears to be better outcomes [22, 54]. This requires further research.

### Limitations

This scoping review was conducted in accordance with a standard framework [33]. This review type was fit-for-purpose, which was to capture a broad understanding of how different training methods impacted on physiotherapists’ appreciation of evidence uptake. Whilst the strength of scoping reviews is constrained by lack of critical appraisal, and lack of independent investigators, the in-depth investigations of training processes provided the required knowledge about how best to train physiotherapy clinicians regarding use of EBP and CPGs.

### Future research

Future studies need to 1) include physiotherapists in the design of training programmes from the conceptual phase, as it may assist in optimising the effect that the programme will have on improving the use of EBP and CPGs; 2) have a robust methodological design with a strong underpinning of behavioural change theory and 3) contextualised the training programme components for the population in which it will be used.

## Conclusion

This review provided a comprehensive framework within which the authors’ novel training programme could be developed for South African physiotherapists using CPGs to put evidence into practice. The training programme will be based on an active KT strategy with multi-faceted interventions, involving physiotherapists as active participants in the learning process. The authors will ensure that they overtly address local contexts, and potential barriers to long term sustainable uptake of EBP and CPGs, with multi-faceted KT interventions. Given the findings of this review, the training programme will also actively seek to measure changes in short and longer-term behaviours and compliance with EBP / CPG practices.

## Additional files

Additional file 1: Articles excluded during initial eligibility assessment, This additional file supplies the reasons for each article being excluded during the initial eligibility assessment. (DOCX 16 kb)

Additional file 2: Articles excluded after applying inclusion and exclusion criteria, This additional file supplies the reasons for each article after applying inclusion and exclusion criteria. (DOCX 14 kb)

## Abbreviations

ALT: Adult learning theory
CPG (s): Clinical practice guideline (s)
EBP: Evidence-based practice
EPOC: Effective Practice and Organisation of Care
IoC: Implementation of change
KT: Knowledge translation
LT: long term
PARiHS: Promoting Action on Research Implementation in Health Services
PE: Professional Education
PT: Physiotherapy
RCT: Randomised controlled trial
SCT: Social cognitive theory
SDL: Self-directed learning
ST: Short term
