# Supplementary material for: Training programmes to improve evidence uptake and utilisation by physiotherapists: a systematic scoping review
Source: BMC Med Educ. 2018 Jan 15;18:14. doi: 10.1186/s12909-018-1121-6 (PMC5769325; doi:10.1186/s12909-018-1121-6)
Supplement: Supplementary file 1 — Articles excluded during initial eligibility assessment, This additional file supplies the reasons for each article being excluded during the initial eligibility assessment. (DOCX 16 kb) [file 12909_2018_1121_MOESM1_ESM.docx]

Additional file 1

Articles excluded during initial eligibility assessment

| Author | Year | Reason |
| --- | --- | --- |
| Willett et al | 2001 | Assessment of individual treatment techniques, not in scope of review |
| Bekkering et al | 2003 | Development of program, not in scope of review |
| Jette et al | 2003 | Development of assessment tool, not in scope of review |
| Evans et al | 2005 | AHP grouped, not in scope of review |
| Valero et al | 2009 | HP grouped, not in scope of review |
| Day et al | 2009 | AHP grouped, not in scope of review |
| Shreiber et al | 2009 | EBP for undergraduate PT, not in scope of review |
| Shreiber et al | 2009 | Formative evaluation study not in scope of review |
| Russel et al | 2010 | Assessing individual outcome measures, not in scope of review |
| Foster et al | 2010 | Protocol of study, not in scope of review |
| Tilson et al | 2010 | Development of assessment tools, not in scope of review |
| McKenzie et al | 2010 | AHP grouped, not in scope of review |
| Evans et al | 2010 | AHP grouped, not in scope of review |
| Horsley et al | 2010 | AHP grouped, not in scope of review |
| Rivald et al | 2010 | Implementation of outcome measures, not in scope of review |
| Maroun et al | 2010 | Implementation of CPGs within an academic institution, not in scope of review |
| Dizon et al | 2011 | Protocol of study, not in scope of review |
| Tilson et al | 2011 | Development of assessment tools, not in scope of review |
| Lewis et al | 2011 | Development of assessment tools, not in scope of review |
| Scott et al | 2011 | Protocol of review, not in scope of review |
| Dizon et al | 2011 | Development of assessment tool, not in scope of review |
| Grimshaw | 2012 | No included PT studies |
| MacDermid et al | 2012 | Protocol of study, not in scope of review |
| Rebbeck et al | 2012 | HP grouped, not in scope of review |
| Lizarondo et al | 2012 | AHP grouped, not in scope of review |
| Demmelmaier et al | 2012 | Assessing prognostic factors, not in scope of review |
| Shreiber et al | 2012 | Use of individual outcome measures, not in scope of review |
| Dizon et al | 2012 | Pilot study followed up with full RCT |
| Campbell et al | 2013 | AHP grouped, not in scope of review |
| Willems et al | 2013 | Protocol of study, not in scope of review |
| Fruth et al | 2013 | Study focussed on PT students, not in scope of review |
| Miller et al | 2013 | Assessment tool testing, not in scope of review |
| Bernhardsson et al | 2013 | Assessment tool testing, not in scope of review |
| Hendrick et al | 2013 | Survey study, not in scope of review |
| Menon | 2013 | AHP grouped, not in scope of review |
| Van Engen-Verheul | 2014 | Protocol of study, not in scope of review |
| Tilson et al | 2014 | Development of program, not in scope of review |
| Shi et al | 2014 | Development of assessment tools, not in scope of review |
| Rutten et al | 2014 | Development of program, not in scope of review |
| Huijg et al | 2014 | Development of assessment tool, not in scope of review |
| Ilic et al | 2014 | Development of assessment tool, not in scope of review |
| Evans et al | 2014 | Not related to implementing EBP or CPGs |
| Fernández-Domínguez et al | 2014 | SR on instruments, not in scope of review |
| Bernhardsson et al | 2014 | Not related to implementing EBP or CPGs |
| Matthews et al | 2015 | KT to patients, not in scope of review |
| Buchanan et al | 2015 | Study on occupational therapists, not in scope of review |
| Harris et al | 2015 | No included PT studies |
| Osteras et al | 2015 | Protocol of study, not in scope of review |
| Imms et al | 2015 | Protocol of study, not in scope of review |
| Gagliardi & Alhabib | 2015 | No included PT studies |
| Maas et al | 2015 | Protocol of study, not in scope of review |
| Fiander et al | 2015 | AHP grouped, not in scope of review |
| Peter et al | 2015 | KT for pt adherence, not in scope of review |
| Camden et al | 2015 | Assessment of specific intervention for pts, not in scope of review |
| O'Brien et al | 2015 | Assessment of intervention to train course givers, not in scope of review |
| Greenfield et al | 2015 | Teaching tools for PT students, not in scope of review |
| Coker-Bolt et al | 2015 | AHP grouped, not in scope of review |
| Rivald et al | 2015 | Education on specific techniques, not in scope of review |
| Stevans et al | 2015 | Case report not in scope of review |
| McElwaine et al | 2016 | AHP grouped, not in scope of review |
| Lin et al | 2016 | HP grouped, not in scope of review |
| Richmond et al | 2016 | KT for pt adherence, not in scope of review |
| Thomas et al | 2016 | Assessment of outcome measure, not in scope of review |
| Connell et al | 2016 | Participatory research study not in scope of review |
| Babatunde | 2017 | Patient adherence assessed, not in scope of review |
