# Supplementary material for: Training programmes to improve evidence uptake and utilisation by physiotherapists: a systematic scoping review
Source: BMC Med Educ. 2018 Jan 15;18:14. doi: 10.1186/s12909-018-1121-6 (PMC5769325; doi:10.1186/s12909-018-1121-6)
Supplement: Supplementary file 2 — Articles excluded after applying inclusion and exclusion criteria, This additional file supplies the reasons for each article after applying inclusion and exclusion criteria. (DOCX 14 kb) [file 12909_2018_1121_MOESM2_ESM.docx]

Additional file 2

Articles excluded after applying inclusion and exclusion criteria

| **Author (year), country** | **Reason for exclusion** |
| --- | --- |
| Hoeijenbos et al (2005), the Netherlands | Assessment of cost effectiveness not in scope of review |
| Rutten et al (2006), the Netherlands | Validation study of outcome measure |
| Stevenson et al (2006), United Kingdom | Article not based on learning how to use EBP or CPG |
| Demmelmaier et al (2012), Sweden | Article not based on the learning of the PTs, rather on caregivers |
| Gross and Lowe (2009), Canada | Implementation of specific treatment recommendations |
| Babatunde et al (2017), Canada | Training PTs to improve pt adherence |
| Schreiber and Dole (2012), USA | Article focussed on use of specific outcome measures |
| Verhoef et al (2004), the Netherlands | PT training to enhance PT services in rheumatology |
| Ketelaar et al (2008), the Netherlands | Article focussed on use of specific outcome measures |
| Russell et al (2010), Canada | Article focussed on use of specific outcome measures |
| Brown et al (2005), USA | Article focussed on use of specific outcome measures |
| Kerssens et al (1999), the Netherlands | Article focussed on use of specific outcome measures |
| Schreiber et al (2009), USA | PT clinical decision making, not in the scope of review |
| Leemrijse et al (2006), the Netherlands | Observational study not in scope of the review |
| Bekkering et al (2005), the Netherlands | Assessment of patient outcomes, not in scope of the review |
| Key: EBP = evidence-based practice; CPG = clinical practice guideline; PT = physiotherapy; pt = patient | |
